# How I use transcranial Doppler in the ICU

**DOI:** 10.1186/s13054-020-2750-9

**Published:** 2020-02-05

**Authors:** Chidinma L. Onweni, Diane C. McLaughlin, William D. Freeman

**Affiliations:** 1 Department of Critical Care Medicine, Mayo Clinic, 4500 San Pablo Rd, Jacksonville, FL 32224 USA; 2 Department of Neurology, Mayo Clinic, 4500 San Pablo Rd, Jacksonville, FL 32224 USA; 3 Department of Neurologic Surgery, Mayo Clinic, 4500 San Pablo Rd, Jacksonville, FL 32224 USA

We read with great interest the article by Drs. Robba and Taccone [1]. We agree completely with the versatility of transcranial Doppler (TCD) ultrasound in the neurointensive care unit (NeuroICU) population and similarly employ it for its uses for aneurysmal subarachnoid hemorrhage, brain death, and circulatory arrest, and as a noninvasive surrogate for intracranial compliance and elastance [2]. We feel the authors’ work is important, especially at a time when TCD seems to be falling out of favor in some NeuroICUs and guidelines. However, we find this ironic, since there is a rise in general critical care ultrasound given its noninvasive utility without ionizing radiation risk compared to computed tomography-based methods of diagnostic evaluation of chest and abdominal body cavities. In our experience, TCD (both blind methods with Spencer ST3 machines, for example) and TCD-imaging methods are useful in the NeuroICU, with 2 major technical caveats: the need for (1) adequate ultrasonographic skills of acquiring imaging and (2) adequate training in interpretation of all features on TCD imaging, including the pulsatility index (i.e., Gosling) and resistance indices (e.g., Pourcelot) [3]. In some cases, teleneurosonology can be performed assuming the 2 caveats above are in place [4]. The most common reason we see for lack of adoption of TCD in intensive care/NeuroICU practice is the lack of these 2 elements. Finally, we note the word “vasospasm” is missing an “s” in Fig. 1, but overall, we commend the authors for their nice summary of TCD use.

## Data Availability

NA

## Abbreviations

NeuroICU: Neurointensive care unit
TCD: Transcranial Doppler
